# Demographic and clinical predictors of unplanned hospital utilisation among chronically ill patients: a prospective cohort study

**DOI:** 10.1186/s12913-015-0789-0

**Published:** 2015-04-03

**Authors:** Kylie-Ann Mallitt, Patrick Kelly, Natalie Plant, Tim Usherwood, James Gillespie, Steven Boyages, Stephen Jan, Stephen Leeder

**Affiliations:** Menzies Centre for Health Policy, University of Sydney, Sydney, NSW Australia; Faculty of Medicine, University of New South Wales, Sydney, NSW Australia; Sydney School of Public Health, University of Sydney, Sydney, NSW Australia; Discipline of General Practice, University of Sydney, Sydney, NSW Australia; The George Institute for Global Health, Camperdown, NSW Australia; eHealthNSW, NSW Health, Sydney, Australia

**Keywords:** Coordinated care, Presentations, Unplanned, Admissions, Emergency department, Health services, Electronic medical record

## Abstract

**Background:**

In urban Australia, patients with serious and continuing illnesses make frequent use of hospital emergency department (ED) services. However, the risk factors for hospital utilisation among the broad population of people with chronic illness are not well known. The aim of this study was to assess the predictors of hospital utilisation (either inpatient admissions or ED visits) in a cohort of 308 patients with chronic illness.

**Methods:**

We studied patients with serious and continuing chronic illnesses presenting to an ED in a large periurban hospital in western Sydney, Australia, between 2010 and 2013. ED presentations and hospital admissions were observed over two years. Multivariate negative-binomial regression analyses were used to identify risk factors for the number of presentations to hospital.

**Results:**

The main risk factors for hospital utilisation were having a live-in carer, and a history of hospital utilisation. Having a live-in carer was associated with an increase in number of ED presentations by 88% (RR 1.88; 95% CI 1.41-2.51), and of admissions by 116% (RR 2.16; 95% CI 1.61-2.92). Seventy-seven percent of hospital utilisation in the cohort was attributable to carer status. Each additional ED presentation that a person had in the 12 months prior to the study led to an increased risk of an ED presentation in the follow-up period by 6% (RR = 1.06, 95% CI = 1.03-1.08). Between 20% and 25% of variability in hospital utilisation in the cohort was attributable to the number of hospital admissions or ED presentations in the previous 12 months.

**Conclusions:**

Patients with a live-in carer and with a history of hospital utilisation are at high risk for future hospital use.

## Background

Chronic illness is characterised by complex causality, a long development period, and a course that runs for years [1]. Globally the burden of illness attributable to chronic disease is massive and rising [2]. Chronic illness now accounts for a greater burden of mortality and morbidity globally than any other groups of disorders including infectious diseases. Cardiovascular disease, mental illness, chronic respiratory conditions, diabetes and cancer contribute to most of this burden, and concurrent multiple conditions are common [3]. Key demographic and clinical risk factors contribute substantially to chronic illness, such as lifestyle factors, socioeconomic status and comorbidities [1].

Globally, chronic illness imposes a heavy financial burden on patients and their families [4], whether publically-sponsored health care is available or not. People with chronic illness use emergency services more frequently, may have longer stays in the emergency department (ED), and are more likely to be admitted [5]. Patients seen in the ED for chronic illnesses are at greater risk of adverse outcomes than all others [6]. Current models of acute emergency care often fail to meet the complex needs of the chronically ill [5], with their multiple comorbidities and medication regimens. To care for these patients adequately requires extended clinical encounter time and allied health professional resources, both of which are often in short supply in busy EDs [7,8].

Health services that avoid ED admissions by preventing exacerbations are considered to be safer, more effective and often more efficient. These services rely on coordination and integration of providers and the delivery of timely and appropriate care for the chronically ill where and when it is needed [9]. Health services may rely on probabilistic analyses and algorithms to better identify and predict those patients who would benefit most from a structured coordinated approach to care. The identification of key elements to include in an effective algorithm for this purpose is highly desirable. This will allow at-risk patients to be targeted for prevention of potential deterioration and admission using coordinated care interventions.

Previous studies examining predictors or risk factors for hospital use and mortality have focused on specific populations (e.g., nursing home, mentally ill, frail elders) [5,10-12] or specific diagnoses (e.g., chronic obstructive pulmonary disease, chronic kidney disease, diabetes, depression) [13-18]. There is currently little knowledge around predictors for hospital use among the broad population of chronically ill patients. In this study, we investigate demographic and clinical predictors of ED presentations and hospital admissions among patients with serious and continuing illness attending a general hospital of 580 beds 65 km west of the central business district of Sydney, Australia.

## Methods

**Figure 1:**
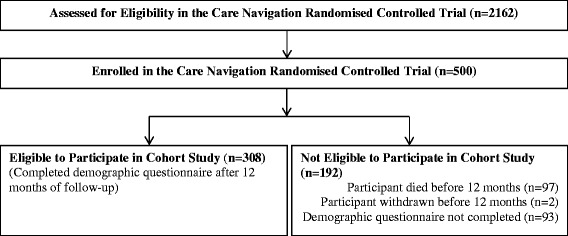
**Flowchart of participants in a prospective cohort study of chronically ill patients in western Sydney, Australia (n, %).**

We conducted a prospective cohort study nested within a randomised controlled trial (RCT) of a within-hospital coordinated care intervention (Care Navigation, or CN), as detailed in Plant *et al*., [19]. Briefly, five-hundred patients were recruited to the RCT and followed for 24 months. The RCT study population was of patients aged ≥70 years with three or more previous hospital admissions in any prior 12 month period (or ≥ 45 years for indigenous patients); and those aged ≥16 with at least one previous respiratory- or cardiac-related hospital admission. Patients are also eligible if a treating clinician determines that a patient would benefit from receiving CN. Of the 500 patients recruited to the RCT, 95 died within 12 months, and 308 were available to complete a phone interview after 12 months follow-up in the RCT. Exclusion criteria for the study were patients with any of the following: previous receipt of CN; medically unable to participate in study, admission to hospital more than 1 CN business day prior to randomisation; or no written informed consent. These 308 patients make up the cohort for this study (Figure 1).

The demographic and detailed clinical characteristics of the patients were collected at 12 months following randomisation. These included age, sex, marital status, language spoken at home, English literacy, carer status, social isolation, source of transport, employment status and education level. Clinical risk factors included body mass index (BMI), smoking status, alcohol consumption, falls history, visual and hearing aide requirement and comorbidities. The number of ED presentations and hospital admissions in the 12 months prior to enrollment into the RCT, and for the 24 month follow up period after enrollment, were collected from a database of Nepean Hospital’s electronic medical record, CERNER.

We assessed the relation of demographic and clinical risk factors to two metrics of health service use: i) number of ED presentations during 24 months’ follow up; and ii) number of hospital admissions during 24 months’ follow up. The association of risk factors with the number of ED re-presentations and hospital admissions was analysed using negative binomial regression, off-set by the length of follow-up time in the study for each patient. Risk factors with a univariate p-value of <0.25 were included in a multivariate model. Backwards step-wise regression was conducted until only significant predictors of each outcome remained. All multivariate statistical models were adjusted for the RCT treatment group (intervention vs. usual care) (results not shown), to take into account the effect of the coordinated care intervention on ED presentations and hospital admissions within the study population. Age and sex were retained in each model, regardless of statistical significance, as these are clinically important characteristics of the target population. Standard assumption-checking and goodness-of-fit analyses were conducted. Population attributable risk percent (PAR) values were calculated to determine the contribution of each statistically significant risk factor to ED presentations and hospital admissions among the target population of chronically ill patients. Multivariate partial PAR values for each risk factor were derived as described in Spiegelman *et al*., [20]. Analysis of variance was used to assess the association between carer status and Urgency, Disposition and Age Grading (UDAG) weight. The UDAG weight is a score of the severity of patient illness in the ED based on age group, disposition (admitted or discharged) and urgency (triage category 1–5).

Written informed consent was obtained from all participants in the study. Ethics approval for this study was obtained from Sydney West Area Health Service Human Research Ethics Committee – Nepean Campus [HREC/09/Nepean/55].

## Results

The median age of the cohort at randomisation was 72.9 years (IQR 63.6 - 80.6; range 32.4 - 91.9). In the 12 months prior to enrolment to the RCT, the median number of ED presentations was 3 (IQR 2 - 5); and the median number of hospital admissions was 2 (IQR 1 - 4).

Descriptive statistics for demographic and clinical characteristics of the study population are shown in Table 1. Nearly half of the patients were male (49.7%), and most were married (48%) or widowed/single (37.2%). Divorced and separated people made up 14.8% of the population. While 277 patients (93.6%) spoke English at home, fewer (72.4%) patients had competent aural, oral and written English literacy. While many patients had a live-in carer (43.6%), over a quarter had no carer (26.6%) and identified as socially isolated (25.3%). The education level of patients was low, with most having only completed primary education (67.5%). Most patients were overweight or obese (62.5%), were former smokers (56.7%), and did not consume alcohol (75.7%). Of those who did consume alcohol, the amount consumed was low (median 3.7 standard drinks per week; IQR 1–7). Functional capacity of patients was moderately impaired; 39.2% had a history of one or more falls in the 12 months prior to being interviewed, 69% had a visual impairment and 46.2% had a hearing impairment.

**Table 1:** **Demographic and clinical baseline descriptive statistics of chronically ill patients in a prospective cohort study in western Sydney, Australia (n, %)**

**Risk factor**	**Categories**	**n**
Sex	Male	153 (49.7)
	Female	155 (50.3)
Marital Status	Married	146 (48.0)
	Separated or Divorced	45 (14.8)
	Single or Widowed	113 (37.2)
Language Spoken at Home	English	277 (93.6)
	Other	19 (6.4)
English Literacy	No	85 (27.6)
	Yes	223 (72.4)
Carer Status	Live-In Carer	133 (43.6)
	Visiting Carer	91 (29.8)
	No Carer	81 (26.6)
Social Isolation	No	230 (74.7)
	Yes	78 (25.3)
Source of Transport	Own Car	121 (40.2)
	Carer	129 (42.9)
	Public or Ambulance	51 (16.9)
Employment Status	Retired	254 (84.4)
	Working/Studying	47 (15.6)
Education Level (completed)	Primary	201 (67.5)
	Secondary	83 (27.9)
	Tertiary	14 (4.7)
Body Mass Index	Underweight	13 (5.1)
	Normal	82 (32.4)
	Overweight	69 (27.3)
	Obese	89 (35.2)
Smoking Status	Never	108 (36.0)
	Former	170 (56.7)
	Current	22 (7.3)
Alcohol Consumption	No	233 (75.7)
	Yes	75 (24.4)
Falls History	No	177 (60.8)
	Yes	114 (39.2)
Visual Impairment	No	93 (31.0)
	Yes	207 (69.0)
Hearing Impairment	No	162 (53.8)
	Yes	139 (46.2)
Heart Disease Comorbidity	No	87 (28.3)
	Yes	221 (71.2)
Respiratory Comorbidity	No	171 (55.5)
	Yes	137 (44.5)
Diabetes Comorbidity	No	204 (66.2)
	Yes	104 (33.8)
Current Cancer Comorbidity	No	284 (92.2)
	Yes	24 (7.8)
Musculoskeletal Comorbidity	No	143 (46.4)
	Yes	165 (53.6)

Of the 308 patients in the follow-up, 39 patients died (12.7%). A total of 1698 ED presentations were observed for 275 patients (89.3% of patients), with a median of four presentations each. The median time to first ED presentation during follow-up was 111 days (IQR 35–270). A total of 1164 hospital admissions occurred for 259 patients (84.1% of patients), with a median of three admissions each. The median time to first hospital admission during follow-up was 143 days (IQR 52–318). For every ED presentation there were 0.68 admissions.

In multivariate regression analysis, significant predictors of the number of hospital admissions during follow-up (Table 2) were female sex (p = 0.0289), carer status (p < 0.0001), respiratory comorbidity (p = 0.0002), no musculoskeletal comorbidity (p = 0.0039), and previous hospital admissions (p < 0.0001). Patients with a live-in carer had 116% more admissions than patients with no carer (RR = 2.16; 95% CI 1.61-2.92). Patients with respiratory comorbidity had 54% more admissions than patients without (RR = 1.54, 95% CI = 1.23-1.94). For every additional hospital admission prior to the study, patients had 9% more admissions during the follow-up period (RR = 1.09, 95% CI = 1.05-1.13). Significant predictors of the number of ED presentations during the 12 month follow-up (Table 3) were female sex (p = 0.0329), divorced marital status (p = 0.0179), whether a person had a carer (p < 0.0001), musculoskeletal comorbidity (p = 0.0046), and previous ED presentations (p < 0.0001). Patients with a live-in carer had 88% more ED presentations than patients with no carer (rate ratio (RR) = 1.88; 95% CI 1.41-2.51). Each additional ED presentation that a person had in the 12 months prior to the study led to an increased risk of an ED presentation in the follow-up period by 6% (RR = 1.06, 95% CI = 1.03-1.08).

**Table 2:** **Results of a negative binomial regression of clinical and demographic risk factors for the number of hospital admissions in a prospective cohort study in western Sydney, Australia (over 24 months)**

		**Number of hospital admissions**			
**Risk factor**	**Reference vs. comparison**	**Univariate rate ratio (95% CI)**	**Univariate p-value**	**Multivariate rate ratio (95% CI)**	Multivariate p-value*
Age	Per 10 years	0.90 (0.81-1.00)	0.053	0.93 (0.84-1.04)	0.194
Sex	Male vs. Female	1.11 (0.87-1.43)	0.400	1.30 (1.03-1.65)	0.0289
Marital Status	Married vs. Divorced	1.90 (1.33-2.70)	0.0006		
	Married vs. Single/Widow	1.01 (0.77-1.32)			
Home Language	English vs. Other	1.05 (0.63-1.74)	0.656		
English Literacy	No vs. Yes	1.10 (0.83-1.45)	0.516		
Carer Status	No Carer vs. Visiting Carer	1.47 (1.05-2.05)	0.0023	1.57 (1.13-2.19)	<0.0001
	No Carer vs. Live-In	1.75 (1.29-2.39)		2.16 (1.61-2.92)	
Social Isolation	No vs. Yes	1.28 (0.97-1.70)	0.083		
Source of Transport	Own Car vs. Carer	1.14 (0.86-1.51)	0.560		
	Own Car vs. Public	1.18 (0.82-1.69)			
Employment Status	Retired vs. Work/Study	1.26 (0.90-1.77)	0.170		
Education Level	Primary vs. Secondary	1.03 (0.77-1.37)	0.911		
	Primary vs. Tertiary	0.90 (0.49-1.63)			
Body Mass Index	Normal vs. Underweight	1.13 (0.59-2.16)	0.584		
	Normal vs. Overweight	1.06 (0.74-1.53)			
	Normal vs. Obese	1.06 (0.75-1.48)			
Smoking Status	Never vs. Former	1.28 (0.98-1.69)	0.0406		
	Never vs. Current	1.78 (1.09-2.92)			
Alcohol Consumption	No vs. Yes	1.09 (0.81-1.45)	0.571		
Falls History	No vs. Yes	1.13 (0.87-1.48)	0.352		
Visual Impairment	No vs. Yes	0.89 (0.68-1.16)	0.383		
Hearing Impairment	No vs. Yes	0.85 (0.66-1.10)	0.212		
Comorbidity	Heart Disease No vs. Yes	1.00 (0.76-1.32)	0.994		
	Respiratory No vs. Yes	1.39 (1.09-1.78)	0.0086	1.54 (1.23-1.94)	0.0002
	Diabetes No vs. Yes	1.30 (0.99-1.68)	0.050		
	Current Cancer No vs. Yes	1.28 (0.77-2.11)	0.336		
	Dementia No vs. Yes	1.40 (0.30-6.58)	0.662		
	Musculoskeletal No vs. Yes	0.69 (0.59-0.84)	0.0039	0.70 (0.55-0.89)	0.0039
Prior ED Presentations	Per Presentation	1.06 (1.03-1.10)	<0.0001		
Prior Admissions	Per Admission	1.11 (1.06-1.16)	<0.0001	1.09 (1.05-1.13)	<0.0001

*All multivariate models are also adjusted for treatment group.

**Table 3:** **Results of a negative binomial regression of clinical and demographic risk factors for the number of ED presentations in a prospective cohort study in western Sydney, Australia (over 24 months)**

		**Number of emergency department presentations**			
**Risk factor**	**Reference vs. comparison**	**Univariate rate ratio (95% CI)**	**Univariate p-value**	**Multivariate rate ratio (95% CI)**	Multivariate p-value*
Age	Per 10 years	0.82 (0.73-0.91)	0.0002	0.96 (0.86-1.07)	0.475
Sex	Male vs. Female	0.96 (0.74-1.25)	0.781	1.31 (1.02-1.66)	0.0329
Marital Status	Married vs. Divorced	2.82 (1.98-4.02)	<0.0001	1.55 (1.12-2.16)	0.0179
	Married vs. Single/Widow	1.04 (0.79-1.38)		1.00 (0.77-1.30)	
Home Language	English vs. Other	0.96 (0.57-1.64)	0.891		
English Literacy	No vs. Yes	1.28 (0.95-1.70)	0.106		
Carer Status	No Carer vs. Visiting Carer	1.74 (1.23-2.45)	0.0040	1.28 (0.92-1.76)	<0.0001
	No Carer vs. Live-In	1.62 (1.18-2.23)		1.88 (1.41-2.51)	
Social Isolation	No vs. Yes	1.06 (0.79-1.43)	0.677		
Source of Transport	Own Car vs. Carer	1.10 (0.82-1.47)	0.0105		
	Own Car vs. Public	1.72 (1.19-2.48)			
Employment Status	Retired vs. Work/Study	1.87 (1.33-2.62)	0.0002		
Education Level	Primary vs. Secondary	0.84 (0.63-1.13)	0.360		
	Primary vs. Tertiary	0.72 (0.38-1.34)			
Body Mass Index	Normal vs. Underweight	1.27 (0.65-2.48)	0.420		
	Normal vs. Overweight	1.18 (0.81-1.72)			
	Normal vs. Obese	1.40 (0.99-1.98)			
Smoking Status	Never vs. Former	1.34 (1.01-1.78)	0.0423		
	Never vs. Current	1.74 (1.04-2.92)			
Alcohol Consumption	No vs. Yes	1.41 (1.05-1.89)	0.0197		
Falls History	No vs. Yes	1.01 (0.77-1.34)	0.917		
Visual Impairment	No vs. Yes	1.07 (0.80-1.42)	0.643		
Hearing Impairment	No vs. Yes	0.69 (0.53-0.90)	0.0064		
Comorbidity	Heart Disease No vs. Yes	0.97 (0.73-1.30)	0.838		
	Respiratory No vs. Yes	1.07 (0.83-1.39)	0.614		
	Diabetes No vs. Yes	1.62 (1.24-2.12)	0.0003		
	Current Cancer No vs. Yes	1.13 (0.67-1.90)	0.638		
	Dementia No vs. Yes	1.04 (0.20-5.32)	0.962		
	Musculoskeletal No vs. Yes	0.57 (0.44-0.74)	<0.0001	0.71 (0.56-0.90)	0.0046
Prior ED Presentations	Per Presentation	1.07 (1.04-1.11)	<0.0001	1.06 (1.03-1.08)	<0.0001
Prior Admissions	Per Admission	1.11 (1.07-1.15)	<0.0001		

*All multivariate models are also adjusted for treatment group.

There was no significant association between UDAG weight and carer status (p = 0.42). The mean (SD) UDAG weight at randomisation for patients with a live-in, visiting, or no carer were 1.5 (0.3), 1.4 (0.3) and 1.5 (0.3), respectively. Population attributable risk percent values are as shown in Table 4 for statistically significant risk factors on i) number of ED presentations; and ii) number of hospital admissions. For both outcomes, between 20% and 25% of variability was attributable to the number of hospital admissions or ED presentations in the previous 12 months. The clinical risk of hospital utilisation was also substantially attributable to carer status (~77% for both outcomes). The population effect of marital status, visual impairment, and comorbidity (musculoskeletal, respiratory and heart disease) was not clinically significant.

**Table 4:** **Attributable risk percent values (and 95% confidence intervals) for the contribution of statistically significant risk factors the number of ED presentations and hospital admissions in western Sydney, Australia (over 24 months)**

	**Number of hospital admissions**	**Number of ED presentations**
Sex	13.3 (2.7, 23.5)	4.5 (0.0, 12.8)
Marital Status	-	0.0 (0.0, 0.0)
Carer Status	77.1 (74.4, 79.5)	77.0 (73.8, 79.8)
Visual Impairment	-	-
Heart Disease Comorbidity	-	-
Respiratory Comorbidity	15.5 (4.4, 26.2)	-
Musculoskeletal Comorbidity	0.0 (0.0, 0.0)	0.0 (0.0, 0.0)
Prior ED Presentations	-	-
Prior Hospital Admissions	20.7 (8.4, 80.7)	24.9 (10.8, 61.6)

## Discussion

This study has identified key predictors of ED presentation and hospital admission in a patient population with chronic illness from western Sydney. The study found being cared for at home and previous ED presentations were associated with increased hospital utilization. The implications are that programs to ensure improved co-ordination of care should be targeted to those living with high levels of dependency and their carers. People with a live-in carer and previous hospital admissions are potentially sicker at presentation to the ED than those without. However, the UDAG weight at randomisation was not different for patients with different carer status. The presence of a live-in carer may facilitate easier access to hospital services; and a live-in carer might recognise deterioration and decide to act on this before the patient would on their own. The presence of live-in carer may also reduce the risk of depression and high psychological distress amongst patients, and may reduce the risk of patients neglecting their own health status. Thus, the increased number of ED presentations and hospital admission among patients with a live-in carer may be due to an increase in necessary hospital utilisation.

Previous studies of acute hospital utilisation among sub-populations of people with chronic illness have identified risk factors for ED presentations and hospital admissions. However, these vary substantially between studies. In particular, age [5,6], recent hospitalisation or ED use [6,21,22], living alone [6,22], lack of social support [6], comorbidities [5,21,22] and overcrowded EDs [23] have been found to predict ED presentations or hospital admissions. Three or more concurrent chronic illnesses have also been shown to predict hospital admissions [5]. However, systematic reviews have shown that most studies find that predisposing factors such as age, sex, and marital status are not predictors of hospital utilisation in the chronically ill. Rather, variables representing psychosocial distress were among the strongest predictors of hospitalisations and physician visits [24,25].

In this study we found no effect of age on ED presentations or hospital admissions, as shown in some previous studies. This is likely because age-related risk factors (such as comorbidities and living arrangements) are significant predictors of hospital utilisation, rather than age *per se.* Also, the majority of patients in the study were 60 or more years so variation in age was markedly constrained.

The strengths of this study were that it was a comprehensive 24 month prospective follow-up of patient outcomes. A limitation was that demographic and clinical risk factors were measured at 12 months into the RCT, rather than at baseline. However, a sensitivity analysis was conducted using data from 12 months post-randomisation to 24 months post-randomisation. The results did not change substantively from those presented here with 24 months of follow-up.

## Conclusion

The identification of risk factors for ED presentation and unplanned hospital admission allows for targeted risk stratification for preventive interventions among the chronically ill. Alternative methods of service delivery, such as large-scale coordinated care, are needed to meet recommended standards for quality health care [25]. Patients with a live-in carer and multiple previous hospital admissions should be targeted for these programs.

## Abbreviations

ED: Emergency department
UDAG: Urgency, disposition and age grading
RR: Rate ratio
RCT: Randomised controlled trial
CN: Care navigation
BMI: Body mass index
PAR: Population attributable risk
IQR: Interquartile range
SD: Standard deviation
